# Individual vs simultaneous macular and optic disc measurements with spectral domain optical coherence tomography in glaucoma and healthy eyes

**DOI:** 10.1038/s41598-024-53293-9

**Published:** 2024-02-01

**Authors:** Abinaya Priya Venkataraman, Loujain Al-Soboh, Johan Hedström, Alberto Domínguez-Vicent

**Affiliations:** Division of Eye and Vision, Department of Clinical Neuroscience, Karolinska Institute, 171 77 Stockholm, Sweden

**Keywords:** Diagnosis, Eye diseases

## Abstract

We assessed the repeatability and agreement of ganglion cell complex (GCC) in the macular area and the peripapillary retinal nerve fiber layer (ppRNFL) with individual and combined macula and disc scans. The macular GCC and ppRNFL thicknesses from 34 control eyes and 43 eyes with glaucoma were measured with the Canon Optical Coherence Tomography (OCT) HS-100. Two repeated measurements were performed with both scan modes. The repeatability limit (Rlim) and agreement analysis were performed. The individual scan showed better repeatability than the combined scan in both groups. However, the differences in the Rlim for the GCC in most sectors were lower than 3 μm (axial resolution of the OCT), and this was larger than 3 μm for most of the ppRNFL sectors. The mean differences in the thickness between both scan modes for the GCC and ppRNFL measurements were less than 3 and 6 μm, respectively. The interval of the limits of agreement was about 10 μm in some sectors for the GCC, and about 40 and 60 μm in some sectors in controls and glaucoma eyes, respectively. Both scan modes showed good repeatability in both groups. The agreement results suggest that the scan modes cannot be used interchangeably.

## Introduction

Glaucoma is characterized by damage to the retinal ganglion cells and their axons, leading to changes in the retinal nerve fiber layer (RNFL) and optic disc and a corresponding loss in the visual function. Optical coherence tomography (OCT) plays a vital role in the detection and management of glaucoma^1,2^. The OCT’s measurements of the macular RNFL, ganglion cell layer (GCL) and peripapillary RNFL (ppRNFL) are the main parameters of interest in glaucoma^3–6^.

The OCT clinically available today have scanning protocols that measure the macula and the optic disc separately as well as combined with a larger scan area. The built-in automated segmentation algorithm provides measures of different retinal layers with these scan modes. The repeatability of the thickness measurements of the macula and optic disc has been well investigated for the commonly used protocols^7–11^. The measurements with wide-field scans have been shown to have similar diagnostic ability to that of the individual macula and optic disc scans in glaucoma detection^12,13^.

The wide scan mode is a common feature in swept course OCTs but some of the spectral domain OCTs also have a larger scan window allowing to image both the macula and optic disc simultaneously. The Canon OCT HS-100 (Canon Europe, the Netherlands), which is a spectral domain OCT, has an updated scanning protocol and segmentation algorithm that performs measurements on an area of 13 × 10 mm, allowing combined analysis of both macula and optic disc. The main difference between the individual macula or disc scans and the combined scans is in the scan resolution which depends on the number of A and B scans. This leads to a larger inter-scan distance in the wide scans, especially for the optic disc evaluation as the individual disc scan has a larger number of A and B scans^14^. The wider interscan distance can affect the repeatability of the thickness measurements.

It is important to know the repeatability and how comparable the thickness values obtained with the simultaneous scanning protocols, which have coarser resolution compared to individual scans with finer resolution. It would also be interesting to know if the repeatability and agreement vary between healthy eyes and in glaucoma, and if it depends on the scan mode. In this study, we evaluated the macular and optic disc thicknesses in healthy eyes and eyes with glaucoma using individual scans of the respective areas as well as with the wide scan mode. We evaluated the repeatability of the ganglion cell complex thickness in the macular area and the ppRNFL thickness with the individual and combined scans as well as the agreement between these scan modes.

## Methods

### Participants

Two groups of participants were included, healthy controls and subjects with glaucoma. The inclusion criteria for the control group were subjects older than 50 years with a best corrected visual acuity better or equal to 0.8 decimal, no history of amblyopia, no history of any ocular surgery, normal anterior and posterior segment (i.e., no significant opacities, irregularities, or pathologies), and intraocular pressure below 21 mmHg. For the glaucoma group, the inclusion criteria were subjects with a previous glaucoma diagnosis (characteristic changes of the optic nerve with corresponding abnormal visual field defects) and no other ocular pathologies.

The study protocol adhered to the tenets of the Declaration of Helsinki and was approved by the Swedish Ethical Review Authority (DRN: 2021-03835). Written informed consent from all participants was obtained after explaining the purpose, nature, and possible consequences of the study.

### Instrumentation and OCT measurements

The Canon OCT HS-100 (Version 4.7.1.0) was used to perform the OCT scans on each participant. This OCT can perform up to 70,000 A-scans/second with an axial resolution of 3 µm and a scan width of up to 13 mm. OCT HS-100 has the possibility to image the macula and optic disc individually (with a maximum scan width of 10- and 6-mm, respectively), or simultaneously with a 13 mm scan width. From this point forward, the term *individual scan* is used to refer to the scan mode that scans only the macular or optic disc, and the term *combined scan* will be used to refer to the scan mode in which the macula and optic disc are imaged simultaneously. In total three different scan modes were performed, and their specifications are summarized in Table 1. Each scan mode was repeated twice. The repeated measurements were taken under repeatability conditions^15,16^, and with sufficient breaks in between to ensure good patient cooperation. The same experienced examiner performed all OCT scans, and these were repeated in case of poor fixation, subject blink, or signal strength less than 7 (out of 10). Only one eye was measured for the control subjects, and one or both eyes were measured for the glaucoma subjects depending on the OCT image quality.

**Table 1:** Specifications of the individual scan and combined scan modes.

	Individual macular scan	Individual disc scan	Combined scan
B-scan orientation	Vertical	Horizontal	Vertical
Number of A-scan	1024	512	512
Number of B-scan	128	256	128
Scan area (mm^2^) horizontal × vertical	10 × 10	6 × 6	13 × 10
Interscan distance, A-scan (μm)	10	12	20
Interscan distance, B-scan (μm)	78	23	102
Parameters evaluated	GCL-IPL RNFL-GCL-IPL	pp RNFL	GCL-IPL RNFL-GCL-IPL pp RNFL

*GCL* ganglion cell layer, *IPL* inner plexiform layer, *RNFL* retinal nerve fiber layer, *ppRNFL* peripapillary retinal nerve fiber layer.

### Parameters analyzed

**Figure 1:**
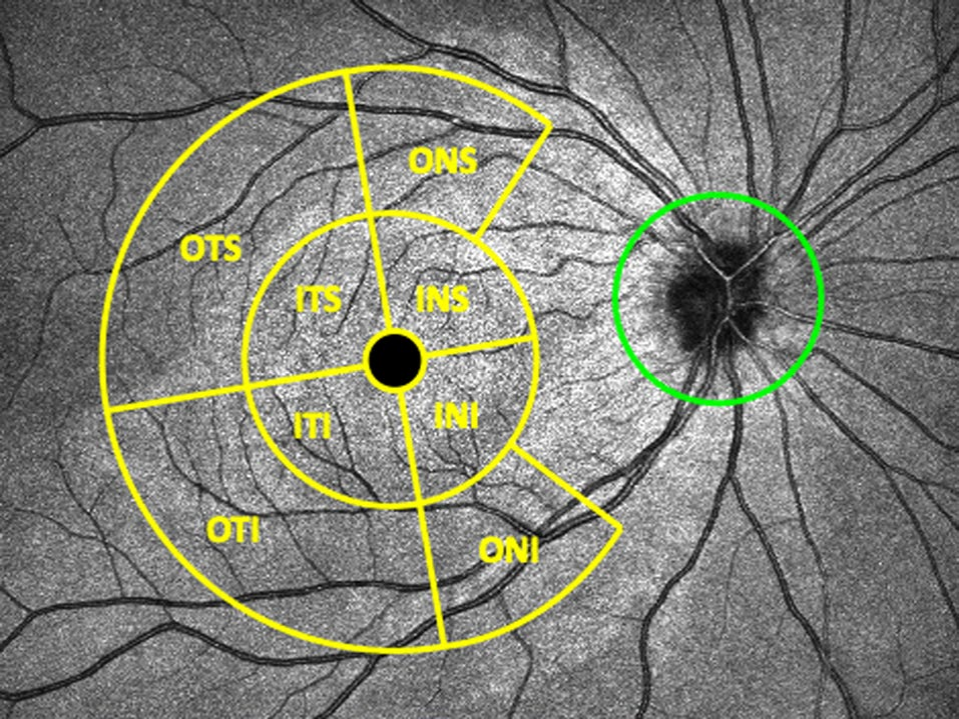
Schematic representation of the thickness map locations overlapped on the scannig laser ophthalmoscopy image from the optical coherence tomography. Yellow grid: ganglion cell complex map. Green circle: Peripapillary retinal nerve fiber layer thickness map. *ITS* inner temporal superior, *INS* inner nasal superior, *INI* inner nasal inferior, *ITI* inner temporal inferior, *OTS* outer temporal superior, *ONS* outer nasal superior, *ONI* outer nasal inferior, *OTI* outer temporal inferior.

From both individual macular and combined scans, the ganglion cell complex thickness was exported. This was measured in terms of retinal nerve fibre layer (RNFL), ganglion cell layer (GCL), and inner plexiform layer (IPL) thicknesses as GCL-IPL and RNFL-GCL-IPL thickness in eight sectors divided into two concentric circles with diameters of 5- and 10 mm. The yellow grid in Fig. 1 shows the ganglion cell complex map location in the macula.

From both individual optic disc and combined scans, the ppRNFL thickness around the optic nerve head was evaluated in twelve clock-hour sectors in a circle of 3.45 mm diameter centred at the optic disc (green grid in Fig. 1). The position of the clock sectors corresponds to the orientation of the right eye. All thicknesses were obtained using the automated segmentation algorithm from the OCT instrument, and no manual adjustments of the segmentation were allowed. However, the centration of the ETDRS map was readjusted manually, if needed.

### Statistical analysis

The baseline demographics of the participants and observations are summarized with descriptive statistics. The within-subject standard deviation (Sw), repeatability limits and coefficient of variation (CoV) were used to describe the repeatability of the OCT HS-100 in both individual and combined scan modes. The Sw, which represents the repeatability of the measurements, was calculated with a one-way analysis of variance. The repeatability limit was calculated as $1.96\cdot \sqrt{2}\cdot {S}_{w}$, and it represents the expected limits that 95% of the measurements should be within^17^. The CoVs were calculated as the repeatability limit divided by the average thickness of that sector and were expressed in percentage.

The Bland–Altman test for repeated measurements was used to analyze the agreement between the individual and combined scan modes^18^. The mean difference between the scans and the 95% limits of agreement are calculated for all the parameters evaluated. The required sample size (n) was determined by considering the standard deviation of the differences between the individual and combined scan modes for the measurement of GCL-IPL from a previous study^14^. Considering the confidence interval of the limit of agreement to be 1 μm, the minimum n value was 34 eyes.

### Ethics approval and consent to participate

The study protocol adhered to the tenets of the Declaration of Helsinki and was approved by the Swedish Ethical Review Authority. Written informed consent from all participants was obtained after explaining the purpose, nature, and the possible consequences of the study.

## Results

A total of 34 healthy controls (7 males and 28 females; mean age: 59.3 ± 6.4 years, range 50 to 72 years) and 27 subjects with diagnosed glaucoma (6 males and 21 females; mean age 74.6 ± 7.4 years, range 47 to 87 years, 43 eyes: 9 mild, 12 moderate and 22 severe glaucoma) were included in this study.

### GCL-IPL thickness

**Figure 2:**
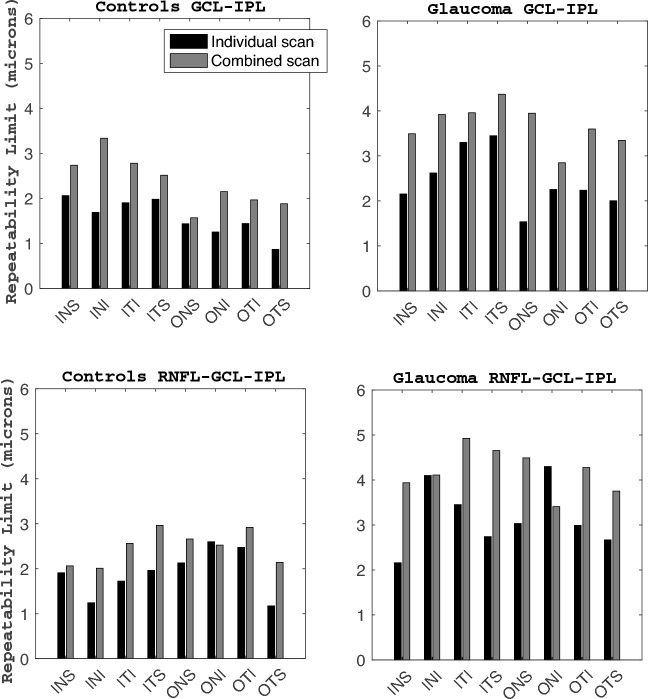
Repeatability limit for the ganglion cell complex thickness measurements for individual and combined scan modes. NFL-GCL-IPL: retinal nerve fiber layer, ganglion cell layer, and inner plexiform layer, and GCL-IPL: ganglion cell layer, and inner plexiform layer. See Fig. 1 legend for sector abbreviations.

**Figure 3:**
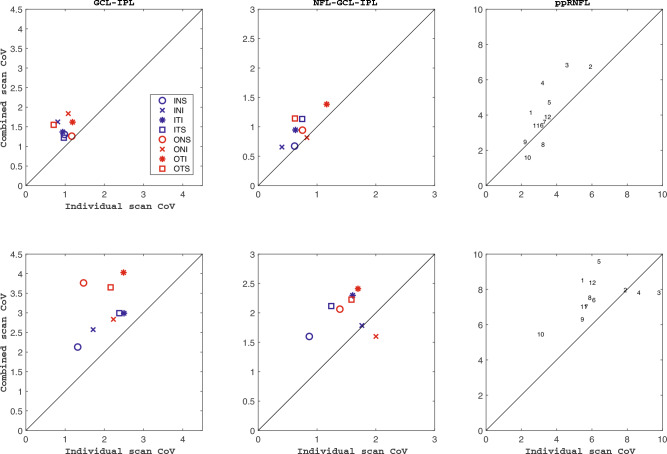
Coefficient of variation (CoV) obtained for the macular and peripapillary retinal nerve fiber layer (ppRNFL) measurements for individual and combined scan modes. NFL-GCL-IPL: retinal nerve fiber layer, ganglion cell layer, and inner plexiform layer, and GCL-IPL: ganglion cell layer, and inner plexiform layer. See Fig. 1 legend for sector abbreviations.

Figure 2 shows the repeatability values for the GCL-IPL (panels A and B) and the NFL-GCL-IPL (panels C and D) thickness. Figure 3 shows the CoVs results for the three thickness maps for the control group (diagrams at the top row) and the glaucoma group (diagrams at the bottom row). The line of equality is represented for visualization purposes. The symbols on top of this line indicate that the combined scan resulted in larger CoV values than the individual scan, and vice versa when the symbols are at the bottom of this line. Table 2 shows the average thickness values for the GCL-IPL and NFL-GCL-IPL thickness of each sector for both population groups and scan modes, and the Bland–Altman results for the comparison between both scan modes.

**Table 2:** Macular ganglion cell complex thickness for 8 different sectors for both scan modes, and the agreement results.

Sector	Sector	Controls			Glaucoma subjects		
		Single scan Mean ± STD	Combined scan Mean ± STD	Mean difference [Limits of agreement]	Single scan Mean ± STD	Combined scan Mean ± STD	Mean difference [Limits of agreement]
GCL-IPL	INS	75.63 ± 6.10	75.75 ± 6.00	− 0.12 [− 2.90 to 2.66]	58.89 ± 11.44	59.22 ± 11.28	− 0.33 [− 4.15 to 3.50]
	INI	75.03 ± 6.11	74.14 ± 6.46	0.89 [− 2.55 to 4.34]	55.19 ± 10.72	55.01 ± 10.80	0.18 [− 4.14 to 4.49]
	ITI	73.32 ± 6.38	73.40 ± 6.32	− 0.08 [− 2.5 to 2.34]	47.63 ± 14.10	47.80 ± 13.98	− 0.17 [− 4.36 to 4.02]
	ITS	73.84 ± 5.82	74.32 ± 5.86	− 0.48 [− 3.08 to 2.12]	52.25 ± 14.38	52.72 ± 13.75	− 0.47 [− 4.89 to 3.94]
	ONS	44.34 ± 3.28	44.98 ± 3.25	− 0.63 [− 2.42 to 1.15]	37.75 ± 5.43	37.88 ± 5.63	− 0.13 [− 3.59 to 3.33]
	ONI	41.93 ± 3.06	42.28 ± 3.38	− 0.36 [− 2.78 to 2.06]	36.46 ± 4.40	36.28 ± 4.60	0.18 [− 3.33 to 3.69]
	OTI	43.76 ± 3.30	43.86 ± 3.30	− 0.10 [− 1.91 to 1.71]	32.43 ± 7.67	32.25 ± 7.42	0.18 [− 3.10 to 3.46]
	OTS	44.13 ± 3.30	43.80 ± 3.33	0.33 [− 1.18 to 1.83]	33.50 ± 7.43	33.08 ± 6.98	0.41 [− 3.10 to 3.92]
NFL-GCL-IPL	INS	111.45 ± 8.91	110.81 ± 8.69	0.64 [− 2.6 to 3.89]	89.79 ± 13.32	88.92 ± 13.84	0.87 [− 2.96 to 4.71]
	INI	111.25 ± 8.48	110.6 ± 8.73	0.65 [− 1.62 to 2.92]	83.96 ± 11.72	83.08 ± 12.00	0.88 [− 4.29 to 6.06]
	ITI	98.42 ± 7.68	97.78 ± 7.77	0.64 [− 1.66 to 2.93]	77.61 ± 10.26	77.39 ± 9.48	0.22 [− 5.38 to 5.82]
	ITS	94.85 ± 6.99	94.395 ± 6.93	0.45 [− 2.29 to 3.19]	79.69 ± 8.97	79.39 ± 9.04	0.31 [− 4.27 to 4.89]
	ONS	102.12 ± 11.55	101.85 ± 9.77	0.27 [− 8.13 to 8.66]	78.83 ± 11.04	78.55 ± 11.22	0.28 [− 4.14 to 4.7]
	ONI	113.31 ± 10.00	111.63 ± 9.82	1.69 [− 5.37 to 8.74]	77.55 ± 15.07	76.89 ± 14.39	0.67 [− 4.13 to 5.47]
	OTI	76.72 ± 6.45	76.19 ± 5.82	0.53 [− 5.73 to 6.79]	63.66 ± 6.62	64.05 ± 6.28	− 0.39 [− 4.98 to 4.2]
	OTS	67.82 ± 5.09	67.72 ± 4.59	0.10 [− 2.2 to 2.41]	60.75 ± 5.00	60.88 ± 4.92	− 0.12 [− 3.75 to 3.5]

All values are expressed in microns.

See Fig. 1 legend for sector abbreviations.

*NFL* retinal nerve fibre layer, *GCL* ganglion cell layer, *IPL* inner plexiform layer, *STD* standard deviation.

Regarding the repeatability metrics for the GCL-IPL thickness, the repeatability limit for the control group ranged from 0.90 to 2.10 μm for the individual scan, and from 1.50 to 3.34 μm for the combined scan; for the glaucoma group, the repeatability limit values ranged from 1.50 to 3.40 μm for the single scan, and from 2.80 to 4.40 μm for the combined scan. Comparing the repeatability limit between both scan modes within the same group and thickness sector map, the individual scan resulted in better repeatability than the combined scan. Nevertheless, these differences were lower than 3 μm. Similarly, the CoVs (Fig. 3) showed the same tendency as all the values are on top of the line of equality for both population groups. The differences in the CoVs between both scan modes were lower than 3% for the control and glaucoma groups. In both groups, the inner sectors resulted in worse repeatability than its corresponding outer sector for the same scan mode. Nevertheless, the CoV values reflected the opposite tendency, where the inner sectors resulted in lower values than the corresponding outer sectors.

The mean difference (Table 2) between the individual and combined scans was lower than 1 μm in each sector. The interval of the limits of agreement for the control and glaucoma group ranged from 3.0 to 7.0 μm, and from 6.5 to 9.0 μm, respectively.

### NFL-GCL-IPL thickness

For the control group, the repeatability limit was lower than 3 μm for both scan protocols. The individual scan protocol showed better repeatability than the combined scan for all sectors except the outer nasal inferior where the difference between both protocols is minimal. The glaucoma group showed a similar tendency as the control group, where the repeatability for the individual scan was better than the combined scan for all sectors but the outer nasal inferior. The repeatability limit for the single scan ranged from 2.2 to 4.3 μm, and for the combined scan it ranged from 3.4 to 5.0 μm. Comparing the repeatability limit between both population groups for the same sector and scan protocol, the glaucoma group showed larger values than the control group. Nonetheless, the differences were lower than 3 μm.

The CoVs (Fig. 3, second column of diagrams) for the control ranged from 0.4 to 1.6%, and the differences between both scan protocols were lower than 1%. The diagram shows that the markers are on top of the equality line, meaning that the CoVs corresponding to the combined scans are larger than the individual scans. On the other hand, the inner sectors (blue markers) showed comparatively lower CoVs than the outer sectors (red markers) except for the temporal superior, where the differences between the inner and outer sectors are minimal.

The CoVs obtained for the glaucoma group ranged from 1.6 to 2.4%, and the differences between the individual and combined scan protocols were lower than 2%. The CoVs for the combined scan are larger than the individual scan protocol for all sectors but one (outer nasal inferior), as all markers are displayed on top of the equality line. The CoVs for the inner sectors were up to 0.5% lower than for the corresponding outer sector for the same scan protocol, except for the nasal inferior sectors.

The mean differences between the individual and combined scan protocols were lower than 2 μm for both population groups. The control group showed that the interval of the limits of agreement for the inner sectors was narrower and more homogeneous than the corresponding outer sectors. Concretely, the intervals ranged from 4 to 6 μm, and from 4 to 17 μm, respectively. For the glaucoma group, the interval of the limits of agreement ranged from 7 to 11 μm for both inner and outer sectors.

### ppRNFL thickness

**Figure 4:**
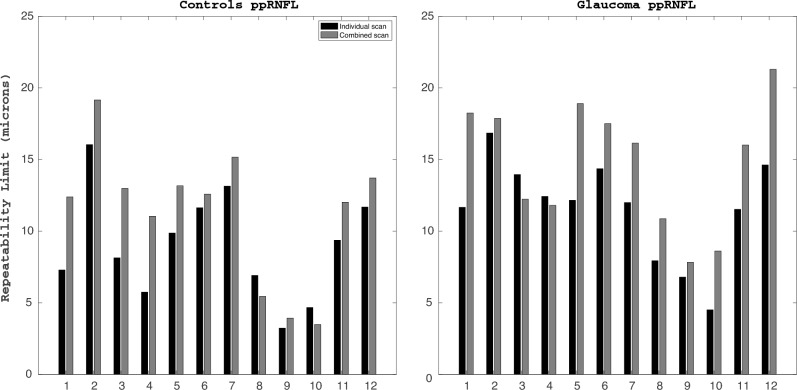
Repeatability limit for the peripapillary retinal nerve fiber layer (ppRNFL) thickness measurements for the 12 clock sectors obtained with the individual and combined scan modes.

The repeatability limit values for the ppRNFL thickness are shown in Fig. 4, and Table 3 shows the average thickness values and the Bland–Altman results for the comparison between both scan modes. Overall, the ppRNFL measurements had worse repeatability than the macular parameters in both groups. In general, the individual scans showed better repeatability than the combined scans for most of the sectors independently of the group. The repeatability limit within the control group was more heterogeneous among the sectors than in the glaucoma group.

**Table 3:** Peripapillary nerve fiber layer thickness for 12 clock sector position with two scan modes, and the agreement results.

Sector	Controls			Glaucoma subjects		
	Single scan Mean ± STD	Combined scan Mean ± STD	Mean difference [Limits of agreement]	Single scan Mean ± STD	Combined scan Mean ± STD	Mean difference [Limits of agreement]
Sector 1	107.93 ± 18.03	108.38 ± 18.44	− 0.45 [− 13.63 to 12.74]	78.41 ± 19.45	77.50 ± 20.19	0.91 [− 19.19 to 21.01]
Sector 2	99.55 ± 14.28	102.61 ± 16.10	− 3.06 [− 23.54 to 17.40]	78.09 ± 19.10	81.12 ± 20.04	− 3.03 [− 23.68 to 17.62]
Sector 3	65.45 ± 10.83	68.66 ± 12.27	− 3.21 [− 20.77 to 14.35]	51.91 ± 14.43	56.53 ± 14.67	− 4.61 [− 17.87 to 8.64]
Sector 4	66.85 ± 13.75	68.65 ± 14.63	− 1.80 [− 13.07 to 9.48]	52.41 ± 13.31	54.55 ± 14.02	− 2.14 [− 18.44 to 14.16]
Sector 5	102.26 ± 19.79	100.73 ± 18.52	1.53 [− 11.03 to 14.09]	69.62 ± 15.93	71.24 ± 16.80	− 1.62 [− 20.41 to 17.17]
Sector 6	137.31 ± 25.97	132.95 ± 24.56	4.36 [− 9.74 to 18.46]	86.22 ± 26.46	85.48 ± 26.75	0.74 [− 21.12 to 22.60]
Sector 7	147.74 ± 16.80	151.96 ± 19.14	− 4.22 [− 23.43 to 14.99]	77.60 ± 24.62	83.11 ± 25.41	− 5.51 [− 34.56 to 23.54]
Sector 8	79.69 ± 12.82	84.22 ± 13.40	− 4.53 [− 14.41 to 5.34]	49.53 ± 13.87	52.16 ± 13.10	− 2.63 [− 15.43 to 10.17]
Sector 9	55.10 ± 6.46	57.13 ± 6.75	− 2.03 [− 6.21 to 2.15]	45.77 ± 11.14	44.85 ± 12.63	0.92 [− 15.45 to 17.30]
Sector 10	78.27 ± 11.68	79.03 ± 11.56	− 0.76 [− 6.12 to 4.61]	56.21 ± 13.90	57.02 ± 13.88	− 0.81 [− 11.33 to 9.71]
Sector 11	127.67 ± 24.87	127.86 ± 23.57	− 0.19 [− 16.94 to 16.56]	77.89 ± 23.15	82.44 ± 21.92	− 4.55 [− 26.61 to 17.51]
Sector 12	128.41 ± 20.13	127.07 ± 21.22	1.34 [− 16.69 to 19.38]	90.65 ± 23.53	91.78 ± 23.67	− 1.13 [− 32.09 to 29.82]

All values are expressed in microns.

*STD* standard deviation.

For the control group, the individual scan showed better repeatability than the combined scan for all sectors except for sectors 8 and 10. It can be also noticed that the repeatability in the vertical sectors was worse than in the horizontal sectors for both scan modes. Concretely, the best repeatability limit for the individual and combined scans was obtained in sectors 9 (3.23 μm) and 10 (3.50 μm), respectively. Both scan modes showed the largest repeatability limit value in sector 2, with a repeatability limit of 16 μm for the individual scan, and 19 μm for the combined scan. The CoVs (Fig. 3) values were on top of the line of equality for all sectors but two (clock position 10 and 8). Nevertheless, sectors 2 and 3 resulted in the largest values for both scan modes.

For the glaucoma group, the individual scan showed better repeatability than the combined scan for all sectors but sectors 3 and 4. Comparatively, the temporal sectors (sectors 8 to 10) showed the best repeatability for both scan modes. Concretely, the values for these three sectors ranged from 5 to 8 μm for the individual scan, and from 8 to 10 μm for the combined scan. For the other nine sectors, the repeatability values ranged from 11.7 to 16.8 μm for the individual scan, and from 11.8 to 21.3 μm for the combined scan. The CoVs (Fig. 3) values were on top of the line of equality for all sectors but two (clock positions 3 and 4). Nevertheless, the variability in the CoV values was larger for the individual scan than in the combined scan. Sectors 2 and 3 resulted in the largest values for both scan modes.

Table 3 shows the descriptive statistics of the ppRNFL thicknesses obtained with each scan modality and the agreement results between the individual and combined scans. On average, the differences between both scan modes were lower than 5 μm for the control group and lower than 6 μm for the glaucoma group. Nevertheless, the interval of the limit of agreement for the control group ranged from 8.46 to 40.94 μm, being minimal in sector 9. For the glaucoma group, the interval of the limit of agreement ranged from 21.04 to 61.91 μm, and it was minimal in sector 10.

## Discussion

The aim of this study was to evaluate the repeatability of the ganglion cell complex thickness in the macula and the ppRNFL thickness with the individual and combined scans in healthy eyes and in eyes with glaucoma. The agreement between these scan modes for each group was also evaluated. In general, the individual scan mode showed better repeatability values than the combined scan mode for the ganglion cell complex in the macular area, and ppRNFL thickness. Nevertheless, the differences were in most sectors lower than the axial resolution of the instrument (3 μm) for the ganglion cell complex. On the other hand, the differences in the repeatability limit were larger than 3 μm for most of the ppRNFL sector map.

A previous study compared the repeatability of a swept-source OCT to measure the ganglion cell complex in the macular area, and the peripapillary RNFL using a 12 × 9 mm (combined) and a 6 × 6 mm (individual) protocols^19^. The results showed that the repeatability and agreement between the scans were high based on the intraclass correlation coefficient values. In a previous study from our group, we showed that the repeatability of the combined scan to measure the ganglion cell complex was up to two times larger than the individual scan in healthy eyes, but never exceeded 6 μm^14^. It should be taken into account that in the previous study the centration of the ETDRS map was not manually adjusted. Whereas, in the present study, the ETDRS map was manually recentered if it was not centered on the center of the fovea automatically. The repeatability of the ganglion cell complex for the healthy controls was in most sectors slightly better than the glaucoma group, irrespective of the scan type. Nonetheless, the differences never exceeded the axial resolution of the instrument.

Though the ganglion cell complex and ppRNFL thickness are shown to have similar diagnostic capabilities in glaucoma detection, ppRNFL has been shown to have a higher diagnostic capability in preperimetric glaucoma cases^5,20,21^. In this study, the repeatability of the ppRNFL thickness measurement showed similar trends with the individual scans showing better repeatability results than the combined scans in general. However, the repeatability exceeded the axial resolution of the instrument in all sectors. It has been documented in the previous literature that the repeatability of the ppRNFL measurements is worse than the ganglion cell complex measurements^8,14^. In this study, there was a difference in the mean age between the groups. As previous studies have shown, age is not a significant confounding factor for achieving reliable OCT thickness data^8,22,23^. Hence, we believe that the age different between the groups does not affect the reliability of the results from the present study.

The main differences between the individual and combined scans are the size of the scan window, scan orientation, and the number of A- and B-scans. This leads to differences in the interscan distances for both the A- and B-scans. For the A-scan, the interscan distance with the combined scan was twice that of the individual scan in both macular and disc areas. However, in terms of B-scan, the differences in the interscan distance were different for the macular and disc areas. The disc area had almost 4 times denser B-scans with the individual scan than the combined scan, whereas in the macular area, it was only 1.3 denser. The scan resolution can affect the repeatability and agreement of the OCT measurements^14,24^. The scan modes used in this study had large differences in the scan resolution. Though the individual scan showed better repeatability than the combined scan, the differences were not that large. Considering the worst repeatability values on each group, it is possible to calculate the number of measurements (N) needed to achieve a defined measurement tolerance using the formula: $1.96\cdot {S}_{w}/\sqrt{N}$. The worst Sw value as such did not differ more than 4 μm among the scan modes and groups. Hence the number of measurements needed to obtain a defined measurement tolerance will be quite similar for each scan mode.

**Figure 5:**
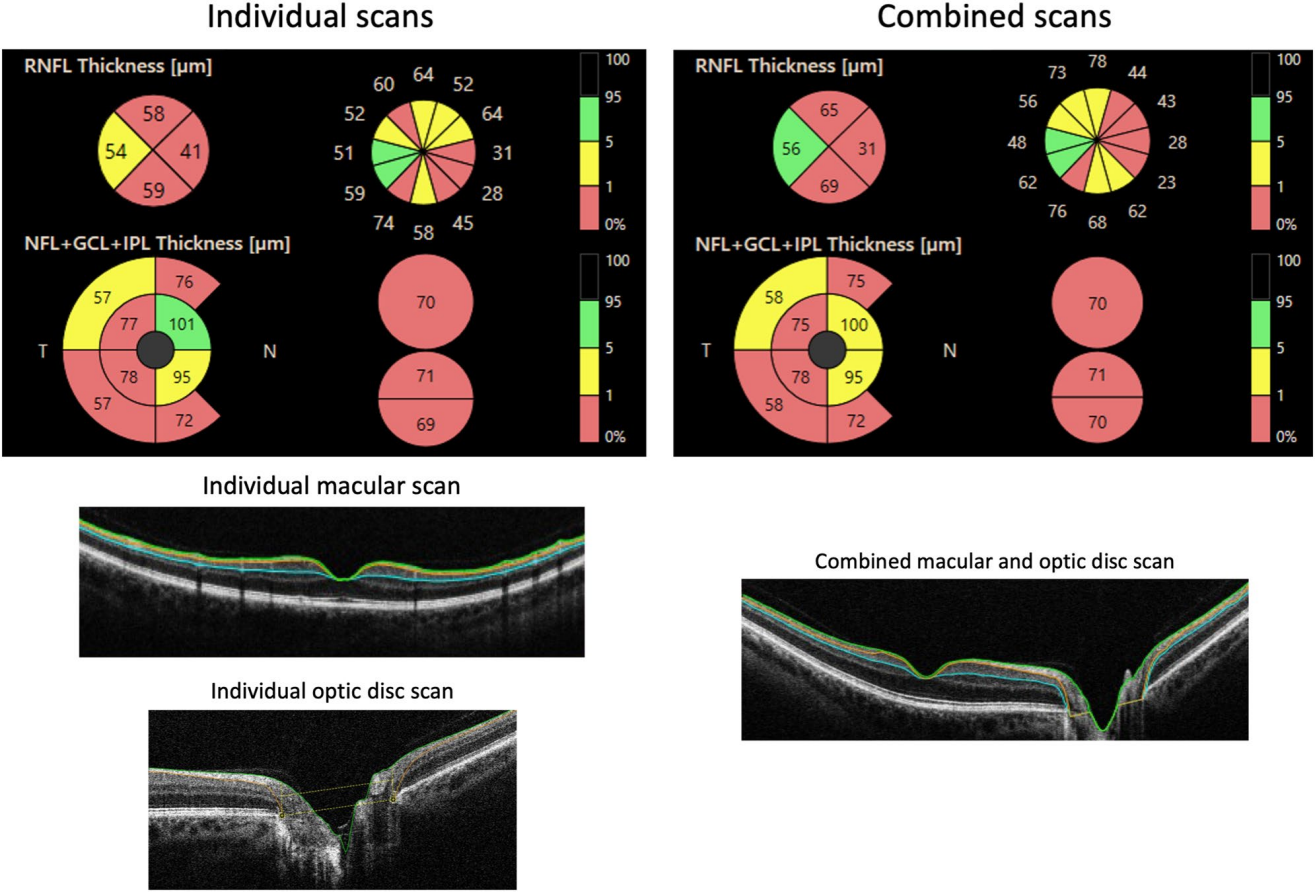
Individual and combined macular and optic disc optical coherence tomography scans from an example glaucoma subject. Top panels show the colour coded map of the peripapillary retinal nerve fiber layer thickness and ganglion cell complex thickness. Bottom panels show example B-scans from the individual and combined scan modes. For abbreviations, refer to Figs. 2 and 3.

Comparing the ganglion cell complex thickness values between both scan modes, the mean differences in the thickness measurement never exceeded the instrument’s axial resolution, and the interval of the limit of agreement was lower than 10 μm in most sectors. However, this is not the case for ppRNFL measurements, where the mean difference was lower than twice the axial resolution, and the limit of the agreement interval was close to 40 and 60 μm in some sectors in healthy controls and glaucoma eyes, respectively. The colour-coded OCT thickness maps obtained with individual and combined macular and optic disc scans from an example glaucoma subject are shown in Fig. 5. It can be seen from this figure that the ppRNFL measurement shows a larger variation between the scans than the ganglion cell complex thickness. This large interval of the limit of agreement in ppRNFL measurements could be mainly due to two factors: the differences in the interscan distances and the scan orientation as the individual scan mode is horizontal by default, whereas the combined scan mode is vertical. From the point of view of repeatability, the performance of the combined scan was quite similar to that of the individual scan. However, considering the agreement results, the measurements of ppRNFL from the two scan modes are not interchangeable in both controls and glaucoma subjects.

In conclusion, the individual and combined scan modes showed good repeatability values, though the healthy controls showed slightly better repeatability than the glaucoma group. The larger interval of the limit of agreement suggests that the scan modes cannot be used interchangeably.

## Data Availability

All data generated or analysed during this study are included in this published article.
